# Effect of Iron Oxide Nanoparticle Incorporation on the Cytocompatibility and Antimicrobial Behavior of a Calcium Silicate-Based Endodontic Sealer

**DOI:** 10.3390/biomedicines14061372

**Published:** 2026-06-18

**Authors:** Riyadh Alshaye, Hanan Alharbi, Wafaa Khalil

**Affiliations:** 1 Endodontic Resident, Department of Conservative Dental Sciences, College of Dentistry, Qassim University, Buraydah 52571, Saudi Arabia; 2 Department of Conservative Dental Sciences, College of Dentistry, Qassim University, Buraydah 52571, Saudi Arabia

**Keywords:** in vitro study, calcium silicate, iron oxide, nanoparticles, materials testing

## Abstract

**Background:** Persistent intraradicular infection and biofilm survival remain major challenges in endodontic treatment, particularly because residual microorganisms may remain within dentinal tubules despite chemomechanical preparation. The antimicrobial efficacy of sealers may be insufficient against resistant bacteria. This study evaluated the effect of incorporating red and black iron oxide nanoparticles into BioRoot RCS on its antimicrobial activity and cytocompatibility. **Methods:** BioRoot RCS was modified with red or black iron oxide nanoparticles at 0.5 wt% and 2.0 wt%, generating 5 groups: unmodified sealer, 0.5% red, 2.0% red, 0.5% black, and 2.0% black. Surface morphology was analyzed using scanning electron microscopy, while elemental composition was determined by energy-dispersive X-ray spectroscopy. Antibacterial activity against *Enterococcus faecalis* and *Fusobacterium nucleatum* was assessed using a direct contact test, antibiofilm activity by colony-forming unit reduction on infected dentin discs, and cytocompatibility using human gingival fibroblasts and the AlamarBlue assay. **Results:** Iron was detected in the modified formulations, and elemental mapping showed homogenous distribution of calcium and iron. The 2.0% formulations showed significantly higher antibacterial and antibiofilm effects than the corresponding 0.5% groups (*p* < 0.05), with 2.0% black showing the lowest bacterial counts. Cytocompatibility differed at 1 and 3 days but not at 7 days, and all groups remained close to the control level with no significant difference (*p* > 0.05). **Conclusions:** Within the limitations of this in vitro study, experimental modification of BioRoot RCS with iron oxide nanoparticles, particularly at 2.0 wt%, improved the antimicrobial and antibiofilm efficacy of BioRoot RCS while maintaining acceptable cytocompatibility. However, physicochemical and handling properties must be evaluated before the clinical relevance of this modification can be determined.

## 1. Introduction

Successful root canal treatment depends not only on chemomechanical debridement, but also on effective sealing of the prepared canal system to entomb residual microorganisms and limit reinfection. This remains challenging because bacteria can persist within dentinal tubules and other anatomical complexities despite instrumentation and irrigation, particularly when organized as biofilms. Biofilm-associated microorganisms exhibit enhanced tolerance to antimicrobial agents and host defenses, making persistent infection a major biologic obstacle in endodontic treatment. *Enterococcus faecalis* is strongly linked to persistent and secondary endodontic infections, while *Fusobacterium nucleatum* is a relevant anaerobic pathogen in primary endodontic infections and a key contributor to biofilm structure and interspecies interactions [1,2,3]. Addressing this challenge requires endodontic materials that combine effective sealing with effective antimicrobial activity.

Calcium silicate-based sealers have been widely used in contemporary endodontics because of their bioactivity, calcium ion release, alkalinizing ability, and generally favorable cytocompatibility [4,5,6,7]. Among them, BioRoot RCS has been reported to show sustained calcium ion release and alkaline pH, features that may support both antimicrobial action and periapical healing [5,6,7,8,9]. However, the magnitude of antibacterial activity varies according to the test model and evaluation time, and the antibacterial performance of conventional calcium silicate-based sealers against biofilm-associated microorganisms remains incomplete. Recent evidence indicates that even among highly alkaline calcium silicate sealers, antibacterial effects may be comparable rather than uniformly superior, suggesting that additional strategies are needed to enhance antimicrobial performance without compromising biologic safety [6,10,11]. This limitation is particularly relevant in biofilm-dominated infections, where established microbial communities are more difficult to eradicate than planktonic cells [1,2,10].

One emerging strategy to address this limitation is nanoparticle incorporation into endodontic materials. Nanoparticles can increase surface reactivity, improve contact with microorganisms, and exert antimicrobial effects through mechanisms such as membrane disruption, oxidative stress, and interference with bacterial metabolism as demonstrated in previously published studies [12]. However, nanoparticle modification is dose-dependent and may alter sealer properties and affect cytocompatibility, making concentration optimization essential. In calcium silicate-based systems, nanoparticle addition has been associated with improved antimicrobial performance and, at appropriate concentrations, maintenance of acceptable cytocompatibility [9,13,14].

Among the nanoparticle systems investigated for biomedical and dental applications, iron oxide nanoparticles (IONPs) are of particular interest because their biological and antimicrobial behavior can vary according to crystalline phase, particle size, surface chemistry, and concentration [15,16]. Red hematite nanoparticles (Fe_2_O_3_) and black magnetite nanoparticles (Fe_3_O_4_) represent two distinct iron oxide formulations with different physicochemical characteristics. Magnetite nanoparticles have been widely investigated in biomedical applications because of their magnetic properties, surface functionalization potential, and generally favorable biocompatibility profile, whereas hematite nanoparticles have also shown antimicrobial potential but may exhibit more dose- and surface-dependent biological responses [15,16,17,18]. These differences make the comparative evaluation of red and black IONPs required when modifying a calcium silicate-based endodontic sealer, especially because any antimicrobial improvement must be balanced against cytocompatibility. However, to the best of our knowledge, the incorporation of IONPs into BioRoot RCS has not been previously reported. Therefore, the aim of this study was to evaluate the effect of incorporating red and black iron oxide nanoparticles at different concentrations into BioRoot RCS on its antibacterial activity and cytocompatibility.

## 2. Materials and Methods

This study was approved by the Institutional Review Board of Qassim University (IRB No. 23-43-20). Extracted human single-rooted teeth were collected from the dental clinics in accordance with institutional requirements. Teeth were selected only if they had no previous endodontic treatment, cracks, root resorption, anatomic abnormalities, or canal calcification. Radiographs were obtained in mesiodistal and buccolingual directions to confirm the presence of a single canal. Teeth were disinfected in chloramine-T (0.5%) and stored at 4 °C for 1 week until use.

### 2.1. Materials

The tested sealer was a calcium silicate-based sealer (BioRoot RCS; Septodont, Saint-Maur-des-Fossés, France; lot no. B33084). Two iron oxide nanoparticle formulations were used: red nanoparticles (Fe_2_O_3_; Sigma-Aldrich, St. Louis, MO, USA; product no. 544884; particle size < 50 nm) and black nanoparticles (Fe_3_O_4_; Sigma-Aldrich, St. Louis, MO, USA; product no. 637106; particle size 50–100 nm).

### 2.2. Nanoparticle Incorporation

The required amount of nanoparticles was weighed using an analytical balance (Radwag, Radom, Poland) and physically blended with the sealer powder in a clean amalgam capsule. The capsule was triturated in an amalgamator (ProMix 2; SDI Limited, Bayswater, Australia) for 30 s. The modified powder was then mixed with the manufacturer’s liquid by combining one scoop of powder with 5 drops of liquid for 60 s.

### 2.3. Experimental Groups

Five groups were evaluated: unmodified sealer (control), sealer containing 0.5 wt% red iron oxide nanoparticles, sealer containing 2.0 wt% red iron oxide nanoparticles, sealer containing 0.5 wt% black iron oxide nanoparticles, and sealer containing 2.0 wt% black iron oxide nanoparticles.

### 2.4. Sample Size

The sample size estimation was determined based on previous in vitro studies with comparable designs and supported by a power analysis. The direct contact antibacterial assay was performed in triplicate for each material group, bacterial species, and exposure time. For the antibiofilm assay, 30 dentin discs were used in total, allocating three discs per group and for each bacterial species. For cytocompatibility testing, 3 independent samples/eluates per group were evaluated, with technical replicates per condition. For SEM/EDX characterization, three specimens per group were examined.

### 2.5. Material Characterization

Freshly mixed sealers were placed in polyvinyl chloride molds (0.8 mm diameter × 2 mm height) and allowed to set at 37 °C and 95% relative humidity for 1 week, then examined using scanning electron microscopy (Quanta 250 FEG; FEI, Eindhoven, The Netherlands). The elemental composition/mapping was analyzed by energy-dispersive X-ray spectroscopy coupled to the same system with SDD Apollo 40 software.

### 2.6. Direct Contact Test

Antibacterial activity was assessed against *E. faecalis* (ATCC 29212) and *F. nucleatum* (ATCC 25586) using a direct contact test. For *F. nucleatum*, all procedures involving bacterial handling, post-exposure recovery, and culture were performed under anaerobic conditions at 37 °C. Briefly, 30 µL of freshly mixed sealer was placed on the side wall of a vertically held 96-well plate, and 10 µL of bacterial suspension (1.5 × 10^6^ cells) was applied directly onto the sealer surface. After 30 or 60 min of incubation at 37 °C and 95% relative humidity, 200 µL of tryptic soy broth was added, the contents were mixed, serially diluted, plated on agar, and colony-forming units were counted after incubation. Each condition was tested in triplicate.

### 2.7. Antibiofilm Assay

Dentin discs were prepared from single-rooted teeth sectioned horizontally at the middle third to obtain 1.5 mm thick slices. A total of 30 dentin discs were prepared, with three discs allocated to each experimental group in addition to the control samples. The canal lumen was enlarged to 2 mm, the discs were cleaned with 5.25% sodium hypochlorite and 17% EDTA, and then sterilized by gamma irradiation. The discs were exposed to *E. faecalis* or *F. nucleatum* suspensions in brain heart infusion broth adjusted to 10^7^ CFU/mL and centrifuged sequentially at 1400, 2000, 3600, and 5600 g, with each step repeated twice for 5 min and fresh bacterial suspension used after each cycle. For *F. nucleatum*, biofilm formation and post-treatment bacterial recovery were performed under anaerobic conditions at 37 °C. The discs were then incubated in fresh brain heart infusion broth for 7 days, with the broth changed every other day. Biofilm formation was confirmed by sonication, vortexing, serial dilution, and culture. Biofilm viability was assessed using the LIVE/DEAD BacLight kit and confocal laser scanning microscopy (Stellaris WLL, Leica, Wetzlar, Germany). After biofilm formation, the assigned sealers were applied directly to the canal lumen of each infected dentin disc. Because the canal lumen diameter and thickness were standardized, the sealer was applied in a standardized manner to completely fill the canal space and ensure direct contact with the infected dentin surface. Excess material was carefully removed from the external surfaces of the disc to maintain comparable material volume and thickness among specimens. The specimens were then incubated and sealer were allowed to set for 7 days at 37 °C and 95% relative humidity with sterile wet gauze to prevent dehydration. After the treatment period, residual viable bacteria were harvested using the same recovery protocol used for biofilm confirmation. Residual biofilm viability was then re-evaluated using the same staining and confocal protocol.

### 2.8. Cytocompatibility Assay

For eluate preparation, 0.3 mL of freshly mixed sealer was placed in each well of a 24-well plate, exposed to ultraviolet light, and covered with 2 mL of growth medium consisting of Dulbecco’s Modified Eagle Medium (DMEM, Gibco BRL, Karlsruhe, Germany), supplemented with L-glutamine, fetal bovine serum, penicillin, streptomycin, and non-essential amino acids. After incubation for 24 h at 37 °C in 5% CO_2_, the eluates were filtered through a 0.2 µm filter, diluted 1:8, and stored at 4 °C until use. Human gingival fibroblasts were seeded in 96-well plates at 10,000 cells/well and exposed to the eluates for 24 h, 3 days, and 7 days. Cell viability was assessed using the AlamarBlue assay, and fluorescence was measured at 560/590 nm using a microplate reader (BioTek Synergy HT, Winooski, VT, USA). Untreated cells served as control, and viability was expressed as a percentage relative to the control.

### 2.9. Statistical Analysis

Statistical analysis was performed using SPSS version 29 (IBM Corp, Armonk, NY, USA). Because the data were not normally distributed, intergroup comparisons were analyzed using the Kruskal–Wallis test followed by Mann–Whitney U tests with Bonferroni correction. Statistical significance was set at *p* < 0.05. Graphs were generated using GraphPad Prism 10 (GraphPad Software, Boston, MA, USA).

## 3. Results

### 3.1. Material Characterization

**Figure 1:**
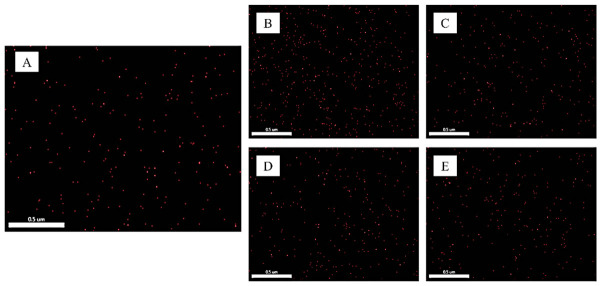
Elemental mapping showing the homogenous iron distribution for (**A**) control sealer. (**B**) 0.5% Red. (**C**) 2.0% Red. (**D**) 0.5% Black. (**E**) 2.0% Black.

**Figure 2:**
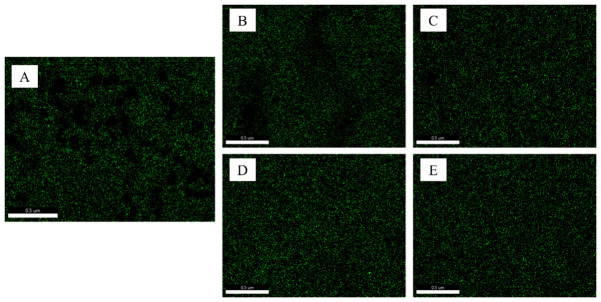
Elemental mapping showing the homogenous calcium distribution for (**A**) control sealer. (**B**) 0.5% Red. (**C**) 2.0% Red. (**D**) 0.5% Black. (**E**) 2.0% Black.

SEM/EDX analysis confirmed the presence of the major constituent elements of the calcium silicate-based sealer in all groups (Table 1), including oxygen, calcium, chlorine, zirconium, and silicon. Iron was detectable in both the unmodified and nanoparticle-modified formulations, with higher iron percentages observed after nanoparticle incorporation. Sodium was detected only in the 2.0% red nanoparticle group. Across the examined specimens, oxygen and calcium remained the predominant elements. Elemental mapping demonstrated homogenous distribution of calcium and iron in the modified groups, supporting the presence and general distribution of iron oxide nanoparticles into the sealer matrix (Figure 1 and Figure 2).

### 3.2. Direct Contact Antibacterial Activity

#### 3.2.1. *Enterococcus faecalis*

**Figure 3:**
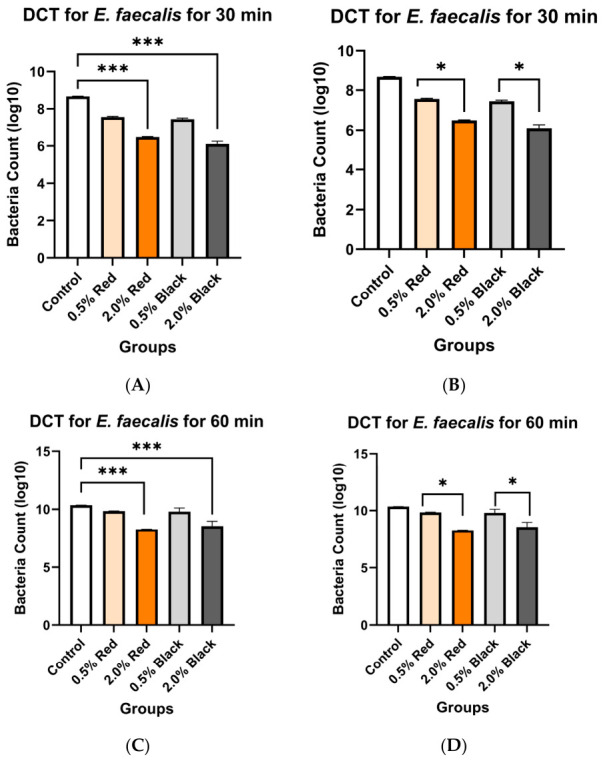
Direct contact antibacterial activity of the tested sealer formulations against *E. faecalis*: (**A**) 30 min, control versus groups; (**B**) 30 min, group-to-group comparisons; (**C**) 60 min, control versus groups; (**D**) 60 min, group-to-group comparisons. (*) means *p* < 0.05. (***) means *p* < 0.001.

At both 30 and 60 min, significant intergroup differences were observed (*p* < 0.05). After 30 and 60 min, both 2.0% formulations showed greater antibacterial activity than the corresponding 0.5% formulations for both black and red groups (*p* < 0.05) and in comparison with the control (*p* < 0.05). No significant difference between the 0.5% red and 0.5% black groups and the control. The 2% Black group exhibited the lowest bacterial count (Figure 3).

#### 3.2.2. *Fusobacterium nucleatum*

**Figure 4:**
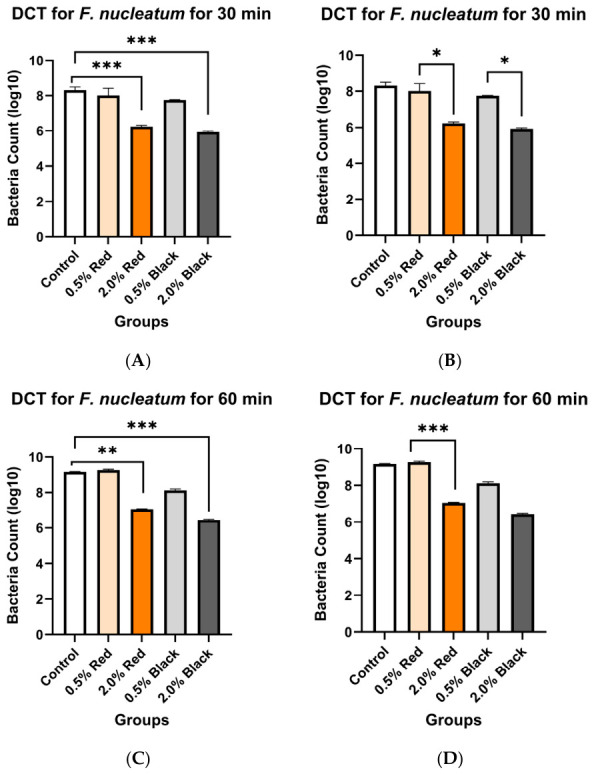
Direct contact antibacterial activity of the tested sealer formulations against *F. nucleatum:* (**A**) 30 min, control versus groups; (**B**) 30 min, group-to-group comparisons; (**C**) 60 min, control versus groups; (**D**) 60 min, group-to-group comparisons. (*) means *p* < 0.05. (**) means *p* < 0.01. (***) means *p* < 0.001.

Similarly, significant intergroup differences were found at both exposure times. At 30 min, the 2.0% red and 2.0% black groups showed significantly lower bacterial counts compared to the control, with 2.0% black again demonstrating the highest antibacterial effect. At 60 min, both 2.0% groups remained more effective than the control and their corresponding 0.5% formulations. Overall, the antibacterial effect against *F. nucleatum* improved with higher nanoparticle concentration (Figure 4).

Comparing *E. faecalis* to *F. nucleatum* after a 30 min exposure shows no significant differences between the groups (*p* > 0.05). However, when comparing the same bacteria after a 60 min exposure, the control and 0.5% Black groups demonstrate significantly better results against *F. nucleatum* (Table 2).

### 3.3. Antibiofilm Activity

**Figure 5:**
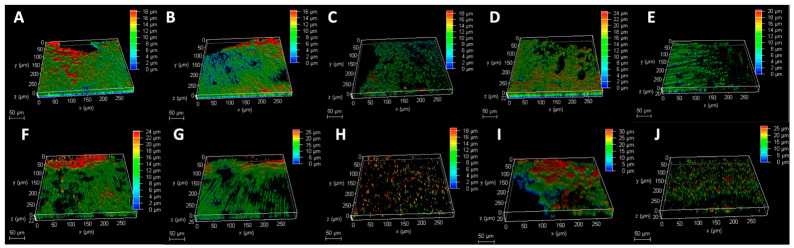
Representative confocal laser scanning microscopy (CLSM) live/dead images of *E. faecalis* and *F. nucleatum* biofilms after treatment with the tested sealer formulations. The upper row shows *E. faecalis* (**A**–**E**), and the lower row shows *F. nucleatum* (**F**–**J**). Images correspond to the control (**A**,**F**), 0.5% red iron oxide nanoparticles (**B**,**G**), 2.0% red iron oxide nanoparticles (**C**,**H**), 0.5% black iron oxide nanoparticles (**D**,**I**), and 2.0% black iron oxide nanoparticles (**E**,**J**).

Antibiofilm activity was interpreted primarily on the basis of CFU reduction (Table 3). Significant intergroup differences were observed for both *E. faecalis* and *F. nucleatum* biofilms (*p* < 0.05). For *E. faecalis*, the lowest CFU values were recorded in the 2.0% black and 2.0% red groups, indicating higher reduction in viable biofilms at higher nanoparticle concentrations (*p* < 0.05). Similarly, for *F. nucleatum*, both 2.0% formulations showed lower viable counts than the control and the corresponding 0.5% groups. Representative Confocal laser scanning microscopy (CLSM) live/dead images visually supported the CFU findings and showed reduced green fluorescence in the higher-concentration groups compared with the control. These images were interpreted only as qualitative visual support and not as an independent quantitative outcome (Figure 5).

### 3.4. Cytocompatibility

Cytocompatibility varied significantly among groups at 1 day and 3 days, but not at 7 days. At day 1, the 0.5% red and 2.0% red formulations showed significantly higher cell viability than their corresponding black nanoparticle groups (*p* < 0.05), although all groups remained close to the control level. At day 3, an overall intergroup difference was still detected. However, no significant differences were found between the treated groups and the control, and no significant differences were observed between corresponding red and black concentrations (*p* > 0.05). By day 7, no significant intergroup differences were found.

Across time, no significant changes were detected between days 1 and 3 for any formulation. Significant temporal differences between days 1 and 7 were observed only in the 0.5% black and 2.0% black groups, whereas between days 3 and 7, only the 0.5% black group showed a significant change (*p* < 0.05). The findings indicate that nanoparticle incorporation did not adversely affect overall cytocompatibility (Table 4).

## 4. Discussion

The present study investigated whether experimental modification of red and black iron oxide nanoparticles into BioRoot RCS could enhance antimicrobial performance while preserving cytocompatibility. Overall, the findings showed that nanoparticle incorporation, particularly at 2.0 wt%, improved the antibacterial activity in direct contact test and reduced viable bacteria in the biofilm for both *Enterococcus faecalis* and *Fusobacterium nucleatum*, and maintained favorable cell viability when tested for cytocompatibility. The findings suggest that iron oxide nanoparticle modification may enhance the antimicrobial profile of BioRoot RCS without compromising cytocompatibility.

BioRoot RCS is a calcium silicate-based sealer that has been increasingly used in contemporary endodontic practice because of its bioactivity, favorable biologic profile, and compatibility with a sealer-based obturation approach [19,20,21,22,23]. Unlike many premixed bioceramic sealers, BioRoot RCS is supplied in a powder-liquid form, which offers a practical experimental advantage for nanoparticle incorporation into the powder phase before sealer mixing. This allows more direct and controlled modification of the sealer formulation than approaches that require post-dispensing incorporation into already reactive pastes [24,25,26]. In the current study, nanoparticle incorporation using physical blending in an amalgamation produced a homogeneous sealer mixture with even IONP distribution. This was confirmed by SEM elemental mapping, a method commonly used in previous studies [27,28]. Based on prior literature, it is repeatedly emphasized that nanoparticle dispersion and agglomeration are critical determinants of whether material modification produces real biologic benefit or simply introduces instability [19,22,24,29].

The improved direct antibacterial activity observed in the 2.0% groups is consistent with the concept that nanoparticle incorporation can support the antimicrobial performance of calcium silicate-based sealers in a concentration-dependent manner. Previous studies on nanoparticle-modified endodontic materials have shown that low-to-moderate nanoparticle loading may enhance antibacterial action, while excessive loading may adversely affect material behavior or biologic compatibility [11,13,14,30]. In the present study, both red and black iron oxide nanoparticle formulations followed the same concept, with the 2.0% groups showing higher antibacterial activity than the corresponding 0.5% groups for both tested microorganisms. This suggests that, within the tested range, increasing nanoparticle content enhanced microbial killing without compromising the overall biologic acceptability of the material. This is in agreement with a previous study that reported a higher antibacterial effect was observed with a higher amount of added Ag nanoparticles [31].

The baseline antimicrobial behavior of BioRoot RCS itself should also be considered when interpreting these findings. Calcium silicate sealers are known to exert antibacterial effects through sustained alkalinization and calcium ion release; these physicochemical changes have been proposed in the literature to contribute to antibacterial activity through mechanisms including bacterial membrane disruption and impairment of metabolic activity, although the precise mechanistic pathways were not directly investigated in the present study [6,8,9,26]. BioRoot sealer has been reported to show favorable calcium ion release and alkaline pH, with antibacterial activity that may persist longer than that of resin-based sealers [6,8,9,10]. However, antibacterial efficacy among calcium silicate sealers is not uniformly superior and may vary considerably depending on the test method, exposure time, and whether bacteria are evaluated in planktonic or biofilm form [8,9,32]. This is clinically relevant because the current study also demonstrated that enhancement through nanoparticle incorporation was more pronounced at the higher concentration, indicating that the intrinsic activity of the parent sealer alone may be insufficient for optimal antimicrobial performance.

The choice of *E. faecalis* and *F. nucleatum* was intended to represent clinically relevant endodontic microorganisms. *E. faecalis* is the most widely used reference organism in endodontic antimicrobial research and is strongly associated with persistent and secondary infections, while *F. nucleatum* is an anaerobic pathogen in primary infections and contributes to interspecies adhesion and biofilm organization [29,33,34]. Evaluating both organisms therefore provided a proper assessment of antimicrobial efficacy across clinically distinct microbial profiles. The observed species-dependent differences at selected exposure periods further support that endodontic antimicrobial evaluation should not rely on a single test organism.

The antibiofilm findings are important and add clinical relevance to the study design. Although direct contact testing is useful for evaluating the effect of materials on planktonic bacteria, biofilm-based assays better reflect the protected and organized mode of microbial growth encountered in root canal infections [1,6,29,31,33,34]. In the present study, antibiofilm activity was interpreted primarily on the basis of CFU reduction, which showed a consistent quantitative outcome across both species. CFU values showed a clear indication toward reduced viable biofilm in the 2.0% groups, especially 2.0% black and 2.0% red. Previous endodontic biofilm studies have similarly relied on a single primary quantitative outcome, such as metabolic activity, viable counts, or live/dead viability ratios, while using microscopy mainly as confirmatory visual evidence [1,3,8].

From a biologic safety perspective, the cytocompatibility results are favorable. Calcium silicate-based sealers are generally reported to be more cytocompatible than epoxy resin-based sealers, with better support for cell viability, adhesion, migration, and mineralization [4,13,20,26,35]. BioRoot sealer is frequently cited among the more favorable formulations, likely because of its calcium silicate chemistry, calcium hydroxide formation, and zirconium oxide radiopacifier profile [4,7,26]. In the present study, all groups maintained cell viability values close to the control level at all tested time points. Although the red nanoparticle groups showed higher viability than the corresponding black groups at day 1, this difference was transient and disappeared over time. Importantly, none of the tested groups showed evidence of marked cytotoxicity. These findings are in line with previous studies indicating that nanoparticle incorporation may remain biologically acceptable at low or moderate concentrations when incorporated into calcium silicate-based matrices, although the balance is highly dependent on nanoparticle type, dispersion quality, and dose [4,24,31,35,36].

Methodologically, AlamarBlue was an appropriate assay for the present cytocompatibility analysis. Compared with some tetrazolium-based assays, fluorescence-based viability assessment may be less susceptible to signal interference in nanoparticle-containing systems, which supports the validity of our cytocompatibility findings [34,35]. This is relevant because assay-material interactions may complicate interpretation when metallic nanoparticles are incorporated into dental materials.

The present study was focused on antibacterial activity, antibiofilm behavior, elemental characterization, and cytocompatibility. However, these findings should be interpreted cautiously. For an endodontic sealer, antimicrobial enhancement alone is insufficient to support clinical use. Essential physicochemical and handling properties, including setting time, flow, film thickness, solubility, radiopacity, pH, dimensional stability and other characteristics, were not evaluated in the present study. These properties may be altered by nanoparticle addition and directly affect obturation quality, material stability, and long-term clinical behavior. Hence, the physicochemical outcomes will be further addressed as a separate component of the broader project. In addition, although SEM-EDX confirmed the presence and general distribution of iron within the sealer matrix, it does not provide definitive evidence of nanoscale dispersion, agglomeration status, chemical interaction, or long-term stability of the nanoparticles within the material. Further characterization using techniques such as TEM, FTIR, XRD, and release/aging analyses would be valuable. Therefore, the present findings should be interpreted as preliminary antimicrobial and cytocompatibility evidence for nanoparticle-modified BioRoot RCS, rather than as evidence of improved overall sealer performance.

## 5. Conclusions

Within the limitations of this in vitro study, experimental modification of BioRoot RCS with red and black iron oxide nanoparticles enhanced its antibacterial and antibiofilm activity, particularly at the 2.0 wt% concentration. Among the modified formulations, the 2.0 wt% black iron oxide nanoparticle group generally demonstrated the most pronounced antimicrobial and antibiofilm effects against *E. faecalis* and *F. nucleatum.* Cytocompatibility with human gingival fibroblasts was maintained over the experimental period, with no significant intergroup differences observed at 7 days. SEM/EDX analysis confirmed the presence and distribution of iron within the modified materials, supporting the feasibility of modifying a calcium silicate-based sealer with iron oxide nanoparticles.

Further studies are needed to determine the effects of this modification on the physicochemical properties and long-term performance before any potential clinical application.

## Figures and Tables

**Table 1:** Elemental composition (wt%) of the tested sealers.

Group	Oxygen (O)	Calcium (Ca)	Chlorine (Cl)	Zirconium (Zr)	Silicon (Si)	Sodium (Na)	Iron (Fe)
Control	43.40%	38.42%	7.57%	8.87%	1.47%	N/A	0.29%
0.5% Red	46%	36.89%	10.96%	5%	0.56%	N/A	0.55%
2.0% Red	48.88%	28.37%	13.56%	5.85%	0.86%	1.99%	0.65%
0.5% Black	42.65%	35.90%	7.71%	10.60%	2.58%	N/A	0.55%
2.0% Black	43.87%	33.21%	15.64%	6.1%	0.73%	N/A	0.61%

**Table 2:** Comparison of the direct contact antibacterial activity of the tested sealer formulations against E. faecalis and *F. nucleatum* at 30 and 60 min (log10 CFU).

Bacteria	Groups									
	After 30 min					After 60 min				
	Control	0.5% Red	2% Red	0.5% Black	2% Black	Control	0.5% Red	2% Red	0.5% Black	2% Black
*E. faecalis*	8.67	7.56	6.49	7.45	6.10	10.35	9.84	8.29	9.81	8.56
*F. nucleatum*	8.31	8.01	6.24	7.76	5.93	9.16	9.26	7.06	8.12	6.44
*p*-value	0.423	0.513	0.788	0.559	0.824	0.037 *	0.490	0.326	0.009 *	0.154

(*) *p* < 0.05.

**Table 3:** Antibiofilm activity expressed as log10 CFU/mL.

Bacteria	Control	0.5% Red	2.0% Red	0.5% Black	2.0% Black	*p* Value
*E. faecalis*	10.14	10.29	9.46	9.88	8.75	<0.001
*F. nucleatum*	10.14	10.26	8.66	9.34	8.60	<0.001

**Table 4:** Cytocompatibility of the tested sealer formulations expressed as percentage cell viability after 1, 3, and 7 days of exposure.

Time	Group					
	Control	0.5% Red	2% Red	0.5% Black	2% Black	*p*-Value
1	100.00	102.43	101.80	97.48	96.39	0.035 *
3	100.00	102.41	104.42	95.60	99.74	0.032 *
7	100.00	99.74	98.99	105.22	102.50	0.433

(*) means *p* < 0.05.

## Data Availability

All data were made publicly available.
